# Experimental Investigation on the Mechanical Performance of Steel-ECC Composite Girders with Corrugated Webs under Negative Moment

**DOI:** 10.3390/ma15196539

**Published:** 2022-09-21

**Authors:** Zhou Fan, Fangwen Wu, Lanqing He, Runbin He, Keyang Zeng, Zhuangzhuang Liu

**Affiliations:** School of Highway, Chang’an University, Xi’an 710064, China

**Keywords:** composite girders, corrugated webs, engineered cementitious composite (ECC), mechanical performance, negative moment

## Abstract

In order to improve the cracking performance in the negative moment region of composite continuous girder bridges with corrugated webs, engineered cementitious composite (ECC) is used instead of conventional normal concrete (NC). Web and concrete types are used as the main research parameters in experiments. The test results indicate that steel-ECC specimens have a higher flexural load capacity and stiffness than steel-NC specimens. The cracks of steel-ECC specimens are characterised by small width and dense distribution. Nonlinear finite element models are established and verified by experimental results. The simulated load–displacement curves are similar to the experimental ones, and the models have a high degree of accuracy. The ECC slab strength, thickness and width are used as parameters for the investigation to analyse the effect of the ECC slab on the flexural bearing capacity of composite girders. Compared with the results of calculations according to the code, the bearing capacity obtained from the parametric analysis is higher. It suggests that the contribution of the ECC slab needs to be considered when calculating the bearing capacity of the steel-ECC composite girder with corrugated webs.

## 1. Introduction

In recent years, with the development of the economy, the demand for bridge construction has increased rapidly. Bridge engineers have attempted to facilitate construction, reduce dead weight, and lower costs. As one of the composite girder bridges, the bridge with corrugated steel webs has the characteristics of lightweight, easy assembly and construction, good durability, and high economic efficiency in the whole life cycle. It is a steel–concrete composite bridge structure type in line with the concept of sustainable development and has a wide range of suitability [1,2,3,4,5,6,7,8].

The mechanical properties of steel–concrete composite girders with corrugated webs have been investigated extensively. Górecki and Sledziewsk [9] fabricated three single-span steel–concrete composite girders with corrugated webs and conducted four-point bending tests to analyse the failure modes, displacement, and strain. The results indicated that bending moment and shear stress were transmitted through the flanges and web, respectively. Elamary et al. [10] investigated the effect of the top flange of steel–concrete composite girders with corrugated webs. It was shown that the steel girder’s top flange significantly affected the structure’s overall performance and affected the concrete slab’s damage mechanism. Lee et al. [11] tested and evaluated the structural performance of prestressed composite girders with corrugated steel webs. Partial interaction was analysed to investigate the horizontal shear transfer mechanism between the steel girder and the concrete. The appropriate arrangement of welded studs could effectively achieve horizontal shear resistance. Zhang et al. [12] introduced a new composite girder with corrugated webs. The top flange consisted of a steel plate and a concrete slab, and prestressed concrete was introduced into the tubular bottom flange. This new composite girder had an adequate bearing capacity, and a simplified formula for calculating the bearing capacity was proposed. Kim et al. [13,14] analysed the flexural performance of prestressed composite girders with corrugated webs by combining theory and tests. The proposed flexural behaviour model was effectively validated, and the horizontal shear strength of the composite girder was accurately evaluated.

Currently, the structural configuration of the composite bridge with corrugated steel webs is mainly the continuous bridge [3,15]. In addition to positive moments, continuous girders also have large negative moments at the supports. However, the current research on composite girders with corrugated steel webs has focused on positive moments and less on negative moments. Under negative moments, the concrete slab is mainly subjected to tensile stress. The concrete slab is prone to cracking, which leads to durability problems and thus affects the overall structural performance.

To solve the problems of concrete cracking and durability, scholars at home and abroad have researched replacing normal concrete (NC) with high-performance concrete in the negative moment region. In recent years, ultra-high-performance concrete (UHPC) has attracted the attention of many researchers due to its high strength and durability [16,17,18]. Zhu et al. [19] investigated steel-UHPC composite girders in negative moment bending tests and compared different joint configurations in the negative moment region. Compared with rectangular joints, T-shaped joints had better resistance to cracking. Liu et al. [20] used UHPC instead of NC to solve the cracking problem of concrete slabs in the negative moment region. A simplified equation was proposed to calculate crack width, and appropriate joint forms and longitudinal laying lengths were suggested. In addition, the mechanical properties of steel-UHPC composite girders were compared with those of steel-NC composite girders [21,22]. The structural failure modes, crack development and deformation characteristics were analysed in detail. It was shown that the former had higher stiffness and better crack control capacity. The mechanical properties of the steel-UHPC composite girders were better than those of the steel-NC composite girders.

Although UHPC has excellent mechanical properties, it requires steam curing to achieve the desired effect. It is often hard to use steam curing in practical projects. Furthermore, the high price of UHPC limits its application [23,24,25]. In recent years, engineered cementitious composite (ECC) has attracted the attention of many scholars due to its strong strain capacity, multiple cracking properties and excellent durability. Both the construction difficulty and price of ECC are reduced compared with UHPC [26,27,28,29,30,31].

Based on these advantages, scholars have attempted to use ECC to solve the problems of NC cracking and durability. Fan et al. [32] investigated the performance of steel-ECC composite girders in the negative moment region. Girders with different concrete slab materials and reinforcement ratios were tested. The stiffness and crack resistance of the structure were improved with the use of ECC. The practical four-parameter fibre-bridging model was proposed to describe the strain-hardening behaviour of ECC. Furthermore, Fan et al. [33] proposed a steel–concrete–ECC composite girder with a hybrid fibre ECC material to obtain a better comprehensive improvement in crack resistance, construction performance and economy. The interface bonding properties between ECC and concrete were good. The analysis of bearing capacity, stiffness and crack development demonstrated the preliminary feasibility and advantages of hybrid fibre ECC for steel–concrete composite girders. Hamoda et al. [34] compared the mechanical properties of different materials in the negative moment region, namely NC, steel fibre-reinforced concrete and ECC. Compared with other specimens, the steel-ECC composite girder had superior cracking characteristics.

Although ECC has been studied in the negative moment region, it has been studied mainly for composite girders with flat webs rather than corrugated webs. Differences in the mechanical behaviour of flat and corrugated webs can result in different structural performance characteristics. Therefore, four specimens were designed to investigate the mechanical properties of the steel-ECC composite girder with corrugated webs under negative moments. Firstly, the failure modes and load-displacement curves of the structure were analysed. Then, the effect of concrete and web types on the structure’s mechanical properties is investigated based on the experimental results. Finally, the influence of the material and dimensional parameters of the concrete slab on the bearing capacity of the steel-ECC composite girder is explored through numerical simulations.

## 2. Experimental Program

### 2.1. Design of Specimens

**Figure 1:**
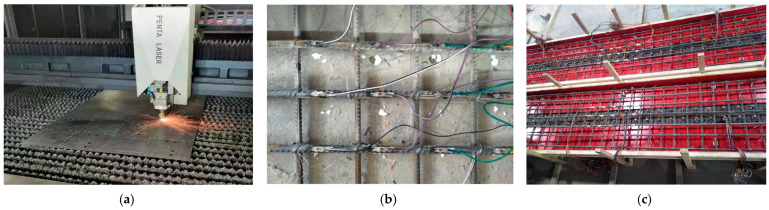
Fabrication of specimens: (**a**) laser cutting; (**b**) strain gauge sticking; (**c**) formwork.

**Figure 2:**
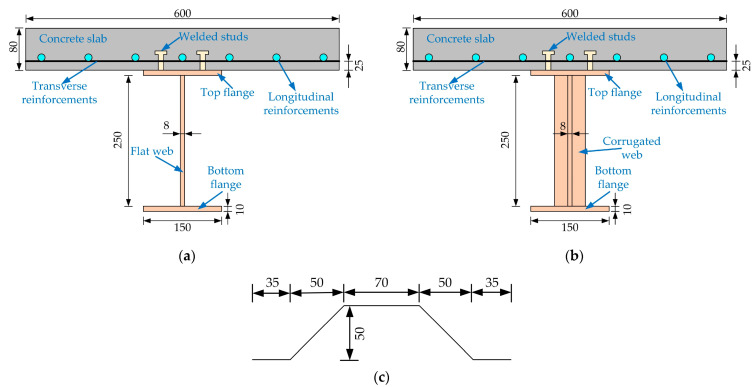
Dimension details of composite girders: (**a**) composite girders with flat webs; (**b**) composite girders with corrugated webs; (**c**) corrugated web profile (unit: mm).

Two steel-NC and two steel-ECC composite girders were designed and constructed, as shown in Figure 1. The main design parameters of the four specimens are illustrated in Table 1. The experimental parameters are the concrete slab type and the web type. Figure 2a,b shows the cross-sections of composite girders with flat and corrugated webs, respectively. The length of the specimen is 3200 mm, and the spring support is 150 mm from the end of the girder.

The concrete slab section dimensions are 600 × 80 mm. The spacing of transverse reinforcements with a diameter of 10 mm is 70 mm. Longitudinal reinforcements of 14 mm diameter are set at a spacing of 90 mm. The flange and web dimensions for all steel girder sections are 150 × 10 mm and 250 × 8 mm, respectively. The composite girders are designed to be fully shear-connected. Welded studs of 10 mm diameter and 40 mm length are welded in 2 rows, with a transverse and longitudinal spacing of 90 and 100 mm.

### 2.2. Material Property Tests

The NC and ECC were commercial concrete produced by Zhongde Xinya Building Materials Co., Ltd., Xinmi, China. Compressive and tensile specimens of the concrete were made to obtain the mechanical properties. In addition, material specimens cut from the steel plate and steel bars were tested. The yield and ultimate strengths were measured and listed in Table 2. The measured properties of NC and ECC are shown in Table 3.

### 2.3. Loading Scheme and Measurements

**Figure 3:**
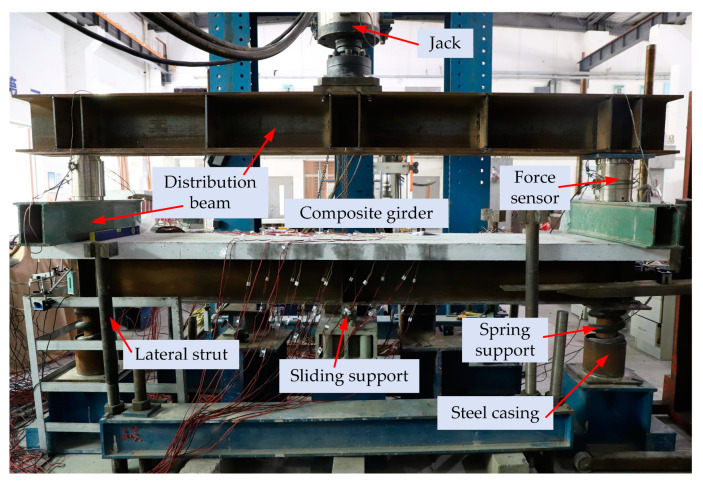
Loading scheme.

In order to simulate the stress more accurately in the negative moment region, the ends of the girder were loaded with springs, as illustrated in Figure 3. This type of loading allowed the observation of crack development and failure modes of concrete slabs. Two-stage distribution beams were used for loading between the jacks and the girders to ensure a uniform distribution of forces. The steel casings were added to the spring supports to avoid out-of-plane deformation, and lateral struts were installed on both sides of the girders. Stiffening ribs were provided at the support and loading points of the girders to avoid local buckling due to stress concentration. Formal loading was carried out using a combination of force control and displacement control. The girders were loaded in increments of 10 kN before cracking and 1 mm after concrete slab cracking until the structures were destroyed.

**Figure 4:**
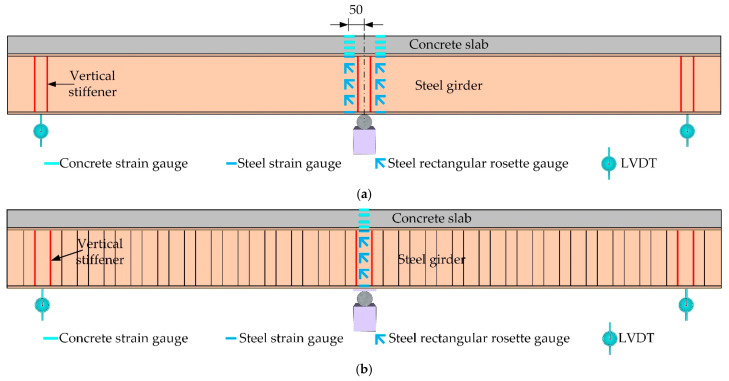
Arrangement of measuring points: (**a**) Composite girders with flat webs; (**b**) Composite girders with corrugated webs (unit: mm).

The measuring points are arranged as shown in Figure 4. Linear variable differential transformers (LVDTs) were used to measure the displacement at the spring supports. Uniaxial strain gauges were used to monitor the forces in the concrete slab and the top and bottom flanges. In order to investigate the shear strain distribution along the height direction of the web, three rectangular rosette gauges were set up on the web.

## 3. Results and Analysis

### 3.1. Experimental Observations and Failure Modes

**Figure 5:**
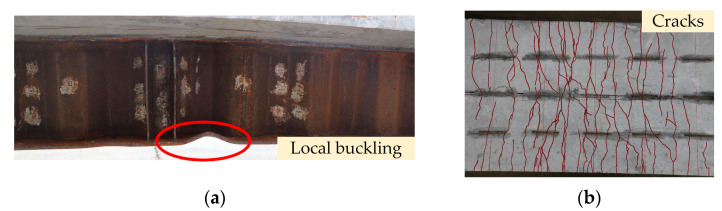
Failure modes: (**a**) local buckling of the bottom flange; (**b**) cracking of the ECC slab.

Four composite girders exhibited bending failure characteristics. The typical failure modes of the girders are shown in Figure 5. The bottom flange was local buckling, and the concrete slab cracked. The steel girder and the concrete slab worked cooperatively and exhibited excellent integrity for all specimens.

For specimen SEC, the first visible crack appeared in the ECC slab near the intermediate support at a load of 55 kN. The crack width increased slowly, but the number increased rapidly. As the loading progressed, the steel bars and bottom flange gradually yielded. When the specimen was damaged, many minute cracks appeared at the top of the slab. Local buckling deformation of the bottom flange was evident and showed a wavy shape. The failure phenomenon of specimens SEC and SEF were similar, except that the cracks of specimens SEF were more densely distributed, and the cracking load was reduced.

The steel-NC specimens and steel-ECC specimens had similar stress on the steel girders, and structural failures were marked by local buckling of the bottom flange. However, the concrete crack development patterns were quite different. The first visible crack appeared earlier in the steel-NC specimens, and the first visible crack appeared in the specimens SNF and SNC concrete slabs at a load of 20 and 15 kN, respectively. The crack width in NC slabs was larger and more widely spaced than in ECC slabs.

### 3.2. Load-Displacement Curves

**Figure 6:**
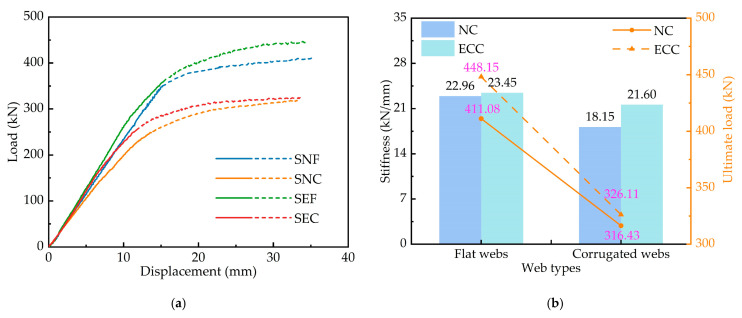
Comparison of specimens: (**a**) load-deflection curves; (**b**) stiffness and ultimate load.

The load–displacement curves of the four composite girders are shown in Figure 6a. It can be seen in the figure that the structures have obvious elastic–plastic characteristics. The solid line in the figure indicates the linear stage of the structure, and the dashed line represents the nonlinear stage. In the initial loading stage, the load and the spring displacement were linear relationships, and the initial stiffness of the four specimens was similar. As the loading proceeded, the concrete cracks gradually developed. The curves remained almost linear up to about 80% of the ultimate load, after which the curves no longer remained linear, the flexural stiffness decreased rapidly, and the structures were damaged. When the load reached about 80% of the ultimate load, the secant stiffness was defined as the specimen stiffness. Figure 6b shows the stiffness and bearing capacity of the four specimens.

ECC and corrugated steel webs could significantly affect the structural performance of steel–concrete composite girders. As seen from Figure 6b, the application of ECC increases the stiffness of composite girders with flat and corrugated webs by 2.11% and 18.98%, respectively, and the flexural bearing capacity by 9.03% and 3.06%. This indicates that ECC could improve the stiffness and bearing capacity of the structure. After NC cracking, the NC slab no longer contributed tensile stress. However, ECC contains a mix of cement, fly ash, sand, water, chemical additive and randomly distributed fibres. The fibres are randomly distributed in the matrix to form a fibre skeleton. After ECC cracking, it can continue to work together by acting as a bridging effect for the fibres. The ECC slab could still maintain large tensile stress due to the bridging action of PVA fibres, which improved the tensile capacity and stiffness of the concrete slab. This also promoted the steel girders’ compressive performance, and the structure could continue to bear the load, thus increasing the bearing capacity.

Compared with flat webs, the application of corrugated webs reduces the stiffness and bearing capacity of the structure on average by 14.42% and 25.12%, respectively. This is mainly due to the “accordion effect” in calculating the stiffness of the corrugated web. The modified elastic modulus of the corrugated web is only 1.09% of that of the flat web, so the axial stiffness of the corrugated web is significantly reduced, resulting in a reduction in the structural stiffness. In addition, the axial stiffness of the corrugated web is negligible and barely bears the bending moments and axial forces. Under negative moments, the compressive stress is mainly concentrated in the bottom flange. The flat web strongly constrains the flanges and plays the role of a stiffening rib without premature buckling and instability. In contrast, the corrugated web has less constraint on the flanges, is equivalent to a slender steel plate and is more prone to instability and buckling under compressive stress, decreasing the structure’s bearing capacity.

### 3.3. Distribution of Strain

**Figure 7:**
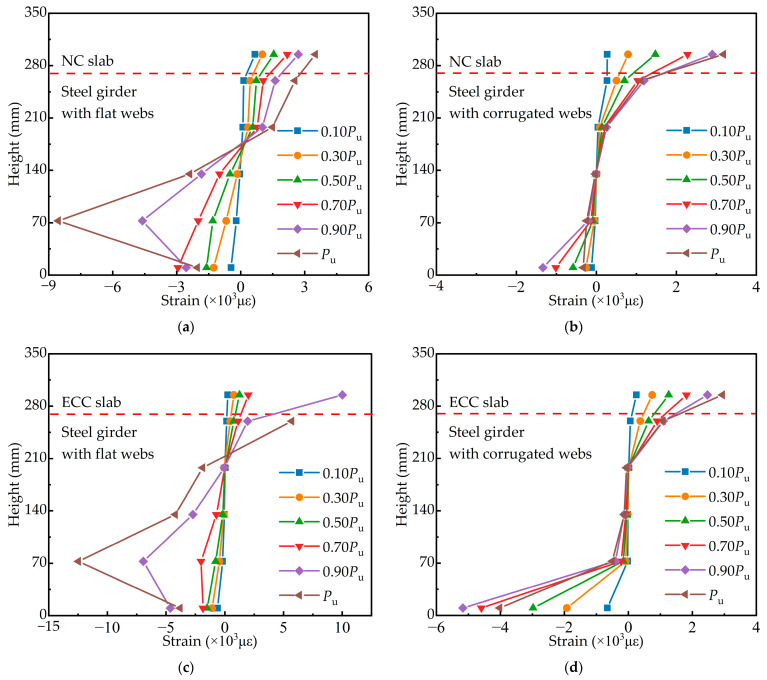
Strain distribution along the height: (**a**) specimen SNF; (**b**) specimen SNC; (**c**) specimen SEF; (**d**) specimen SEC.

The strain distribution along the height direction of the measured section is plotted in Figure 7. The dashed line indicates the interface of the steel–concrete composite girder. The upper part of the dashed line is the concrete slab, and the lower part is the steel girder. The figure shows that the top flange of the steel girder is in tension and the bottom flange is in compression, with the neutral axis inside the web. There is a noticeable difference in the strain distribution between composite girders with flat and corrugated webs. The strain distribution along the height of the section is essentially linear for composite girders with flat webs, in line with the “plane section assumption”. However, due to the use of corrugated webs, the strain distribution in the steel girder section deviates significantly from linear. Suppose the strain of the corrugated webs is not considered, and other strain measurement points are connected. In that case, the strain distribution at these points is almost linear, following the “quasi plane assumption” [35], which can form the basis for calculating the flexural bearing capacity.

**Figure 8:**
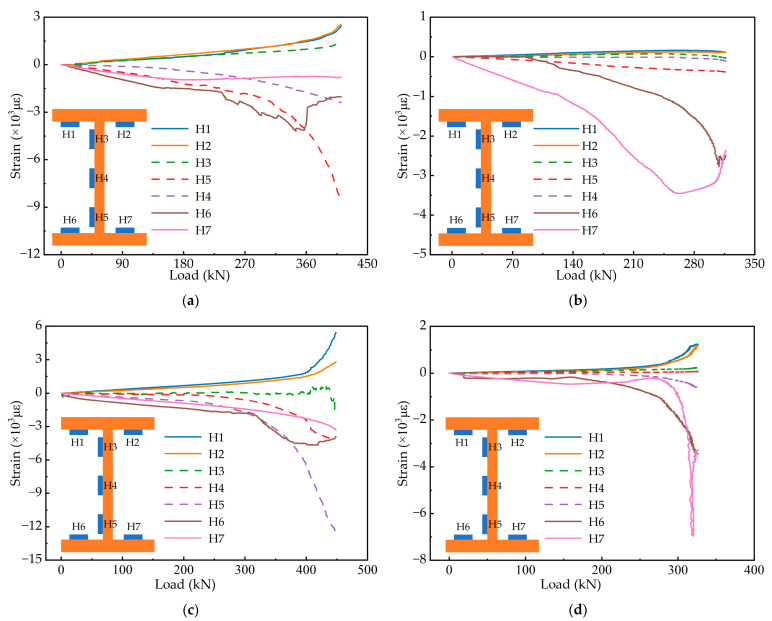
Strain development of the steel girder: (**a**) specimen SNF; (**b**) specimen SNC; (**c**) specimen SEF; (**d**) specimen SEC.

The strain variation of the steel girder during loading is shown in Figure 8. The dashed lines denote the measurement points on the web, and the solid lines indicate the measurement points on the flanges. Due to the “accordion effect”, the flanges of the corrugated web specimens are mainly subjected to axial strain, and the strain of the web is minimal. The top flange is mainly subjected to tensile stress, and the strain of the top flange increases rapidly during the late loading. The compressive strain on one side of the bottom flange increases rapidly, and on the other side, the compressive strain increases to a certain level and then decreases quickly, even with tensile stress. The bottom flange buckles under the effect of the larger compressive stress. In Figure 8b, the strain of H6 and H7 sensors is all measured values. In analysing the data, we found a certain deviation between the measured value of H6 and the actual value, which is caused by the problem with the strain gauge. Considering that the trend of the data is basically in line with reality, and in order to maintain the integrity and authenticity of the data, we retain the data and draw it in Figure 8b.

As shown in the diagram, ECC and corrugated webs affect the strain of the steel girder. With excellent tensile properties, ECC could still exert a tensile effect at the late loading stage. ECC improves the tensile properties of the concrete slab and can withstand more tensile stress, allowing the performance of the steel girder to be more fully exploited. As a result, the steel girder strain of the steel-ECC specimens is larger than that of the steel-NC specimens. However, the maximum strain of the steel girder with corrugated webs is only about 50% of that with flat webs. This is because the composite girder with corrugated webs is more susceptible to instability under the negative moment than the composite girder with flat webs, resulting in lower bearing capacity and reduced girder strain.

### 3.4. Distribution of Shear Stress

In this section, the shear strain behaviour of the web of composite girders is analysed. The shear strain *γ* at each measurement point was calculated according to the rectangular rosette gauges on the web. The calculation equation is as follows:

(1) $$\gamma =\sqrt{(\varepsilon_{0}-\varepsilon_{90}{)}^{2}+(2\varepsilon_{45}-\varepsilon_{0}-\varepsilon_{90}{)}^{2}}$$

Here, *ε*_0_, *ε*_45_ and *ε*_90_ indicate the strain in the three directions on the rectangular rosette gauges, and the subscripts indicate the angle between the rosette gauge and the horizontal direction. The following equation calculates the shear stress *τ* of the measuring point:

(2) τ=Gγ

where *G* is the shear modulus. However, the shear modulus of corrugated webs is different from that of flat webs and needs to be calculated by the following equation [36]:

(3) $$G_{c}=\frac{a+b}{a+c}\frac{E}{2(1+\upsilon )}$$

where *G*_c_ is the equivalent shear modulus, *a* is the flat panel length, *b* is the horizontal projection of the inclined panel width, *c* is the inclined panel length, and *υ* is the Poisson ratio of corrugated webs.

**Figure 9:**
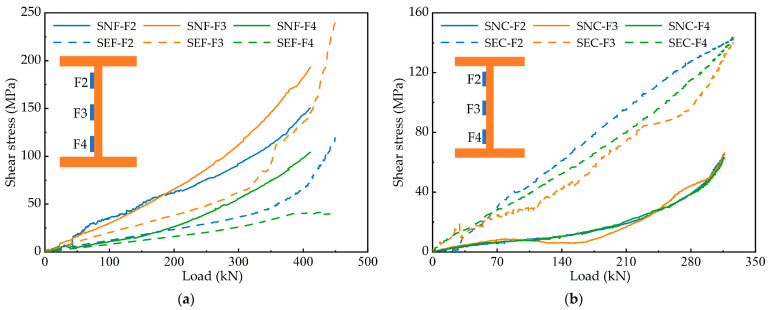
Effect of different concretes on shear stress: (**a**) specimens with flat webs; (**b**) specimens with corrugated webs.

Figure 9 shows the variation of shear stress for each specimen. It is evident from the figure that the shear stress distribution pattern of corrugated webs is different from that of flat webs.

The shear stress of flat webs is unevenly distributed along the height direction, showing the characteristics of small at both ends and large in the middle. The shear stress distribution of corrugated webs is uniform, and the load–shear stress curves are similar. The stress state of steel girders with corrugated webs is comparable to that of trusses. The top and bottom flanges are equivalent to chords, and corrugated webs are equivalent to web members. Corrugated webs act as diagonal web members through the tension field. Corrugated webs bear almost no bending moment; therefore, the shear stress of the webs is evenly distributed. In addition, using ECC to increase the bearing capacity also increases the shear stress, and the shear stress of the steel-ECC specimens is significantly larger than that of the steel-NC specimens.

The above analysis makes it not difficult to see that corrugated webs bear virtually no bending moment. The bending moment is borne by the concrete slab and the top and bottom flanges of the steel girder, and the corrugated webs are mainly shear-resistant. The division of labour between the two is clear.

### 3.5. Crack Analysis

**Figure 10:**
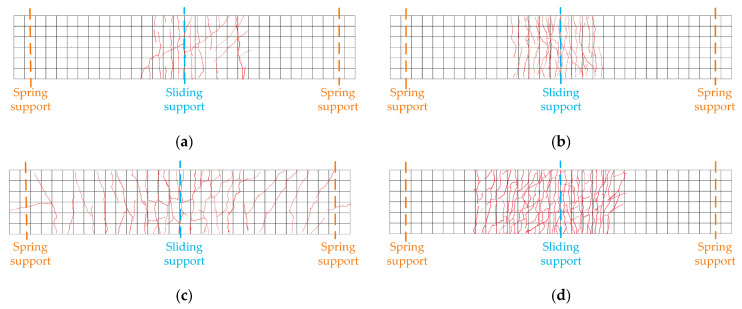
Crack development at top of the slab: (**a**) specimen SNF; (**b**) specimen SNC; (**c**) specimen SEF; (**d**) specimen SEC.

The crack distribution of each specimen is plotted in Figure 10. The cracks in the specimens were all mainly concentrated in the area around the intermediate support. The first crack in the specimen occurred in the area of the intermediate support. Subsequently, several cracks appeared on both sides of the intermediate support. As the load increased, the cracks gradually expanded, increasing in length and width.

The specimen SEF exhibits a characteristic of showing multiple cracks with a dense distribution. The main reason for the phenomenon is that the PVA fibres between the cracks play a bridging role after cracking. The fibres restrain the matrix on both sides away from the cracks, keeping cracks slim and limiting the expansion of cracks. This reflects the excellent crack control capacity of ECC. The incorporation of PVA fibres also increases the cracking load of the structure.

However, the crack characteristics of NC slabs differ significantly from those of ECC slabs. After NC slab cracking, the cracks extended from the top to the sides. Eventually, NC slabs quit working. Although the crack width of NC slabs grew faster than that of ECC slabs, the number of cracks of ECC slabs increased more rapidly. The steel-NC specimens had larger crack spacing and wider cracks. The crack expansion pattern of specimens with corrugated webs was similar to that with flat webs. Nevertheless, due to the existence of accordion effect, the corrugated web is weakly restrained to the upper part, which is challenging to limit the crack expansion. The crack spacing and width of specimens SNC and SEC were larger than specimens SNF and SEF.

### 3.6. Comparison with Current Codes

In calculating the flexural bending capacity in the negative moment region of the composite girder with corrugated webs, the Chinese code CECS 291:2011 [37] ignores the contribution of the concrete slab. This is because NC cannot continue to bear tensile stress after cracking. The calculation of the flexural bending capacity in the code is as follows.

(4) $$M=A_{\mathrm{st}}f_{\mathrm{st}}(y+h_{s})+A_{\mathrm{tf}}fh_{s}$$

In the formula, *A*_st_ and *f*_st_ are the steel bars’ cross-sectional area and tensile strength, respectively. *y* denotes the distance from the cross-sectional centroid of the longitudinal steel to the centroid of the top flange of the steel girder. *A*_tf_ represents the cross-sectional area of the top flange, and *h*_s_ is the distance between the centroids of the top and bottom flanges. *f* denotes the tensile strength of the steel plate.

The code calculated and measured values for the steel-ECC composite girders with corrugated webs are summarised in Table 4. The flexural bearing capacity of the composite girder is all higher than the code calculated values. It indicates that the code calculations are conservative, considering only the steel bars and steel girder flexural resistance and ignoring the concrete slab contribution. The error of bearing capacity of specimen SEC is greater than that of specimen SNC. This is because ECC can exhibit ultra-high tensile strain through the bridging action between the fibres. It can still exert a tensile effect after cracking, increasing the bearing capacity. In conclusion, the contribution of ECC slabs to the flexural bearing capacity in the negative moment region is evident, which needs to be considered in code calculations.

## 4. Numerical Investigation

### 4.1. Finite Element Model

**Figure 11:**
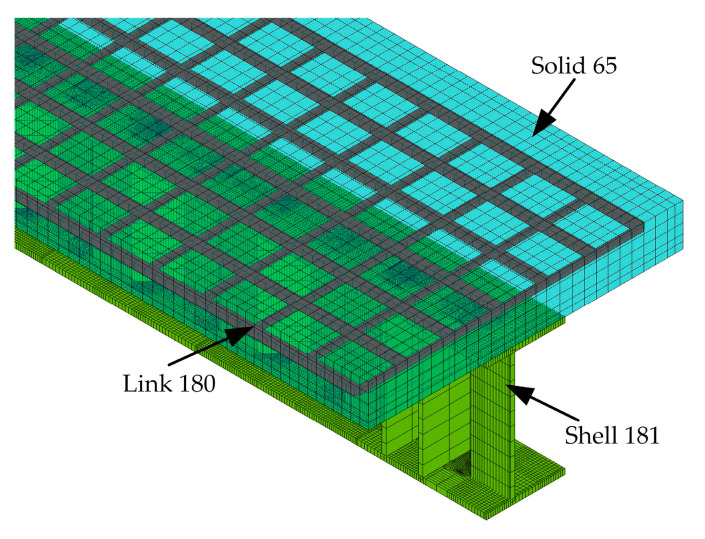
Finite element model.

In order to further investigate the mechanical behaviour of composite girders, the three-dimensional models are established by ANSYS, as shown in Figure 11. The concrete slab is modelled using the 3D eight-node solid element (Solid 65) with a mesh size of 25 mm. The steel girder and stiffening rib are modelled using the four-node shell element (Shell 181), and the mesh size is 25 mm. The mesh refinement is used for local positions of the flanges. The composite girders are the complete shear connection, and the welded studs are not damaged under ultimate load. Therefore, the welded studs can be simulated using the nonlinear unidirectional spring element (Combine 39). The 3D link element (Link 180) is used to simulate the reinforcement in the concrete slab, which is embedded in the concrete slab, ignoring the relative slip between the two. The Solid 185 element is adopted to simulate the steel cushion block to better simulate the actual loading and boundary conditions.

**Figure 12:**
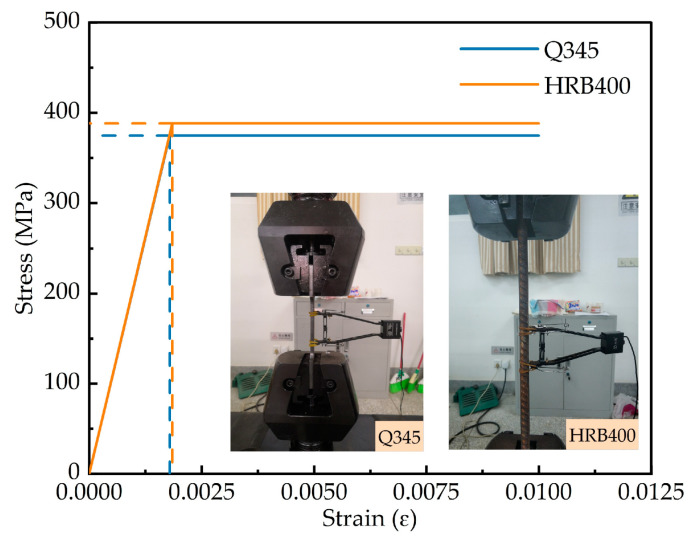
Constitutive model of steel and steel bars.

The nonlinearity of the structure is considered in the numerical analysis. As shown in Figure 12, the ideal elastic–plastic material model is adopted for steel and steel bars, respectively. The NC stress–strain relationship is selected according to the GB 50010-2010 [38]. The ECC constitutive relation adopts the stress–strain relation curve proposed by Yuan et al. [39], which is shown in Equation (5) for compression and Equation (6) for tension. The William–Warnke five-parameter failure criterion is used to define the concrete failure criterion. The actual performance parameters of various materials are obtained through material properties tests.

(5) $$\sigma_{c}=\left\{ \begin{array}{ll} 2\frac{\sigma_{c0}}{\varepsilon_{c0}}\varepsilon & 0\leq \varepsilon \leq \frac{\varepsilon_{c0}}{3}\\ \frac{\sigma_{c0}}{2\varepsilon_{c0}}\varepsilon +\frac{\sigma_{c0}}{2} & \frac{\varepsilon_{c0}}{3}<\varepsilon \leq \varepsilon_{c0}\\ -\frac{\sigma_{c0}}{\varepsilon_{c0}}\varepsilon +2\sigma_{c0} & \varepsilon_{c0}\;<\varepsilon \leq \frac{3\varepsilon_{c0}}{2}\\ \frac{\sigma_{c0}}{3\varepsilon_{c0}-2\varepsilon_{\mathrm{cu}}}(\varepsilon -\varepsilon_{\mathrm{cu}}) & \frac{3\varepsilon_{c0}}{2}<\varepsilon \leq \varepsilon_{\mathrm{cu}} \end{array}\right.$$

where *σ*_c0_ is the axial compressive strength, taken as 50.3 MPa; *ε*_c0_ is the peak axial compressive strain, the value of 0.00405; *ε*_cu_ is the ultimate compressive strain, as 0.0115.

(6) $$\sigma_{t}=\left\{ \begin{array}{ll} \frac{\sigma_{\mathrm{tc}}}{\varepsilon_{\mathrm{tc}}}\varepsilon & 0\leq \varepsilon \leq \varepsilon_{\mathrm{tc}}\\ \frac{\sigma_{\mathrm{tu}}-\sigma_{\mathrm{tc}}}{\varepsilon_{\mathrm{tu}}-\varepsilon_{\mathrm{tc}}}(\varepsilon -\varepsilon_{\mathrm{tc}})+\sigma_{\mathrm{tc}} & \varepsilon_{\mathrm{tc}}<\varepsilon \leq \varepsilon_{\mathrm{tu}} \end{array}\right.$$

where *σ*_tc_ is the cracking strength, taken as 3.96 MPa; *ε*_tc_ is the cracking strain, for 0.000215; *σ*_tu_ is the ultimate tensile strength, as 4.63 MPa; and *ε*_tu_ is the ultimate tensile strain, that is 0.02.

### 4.2. Model Validation

**Figure 13:**
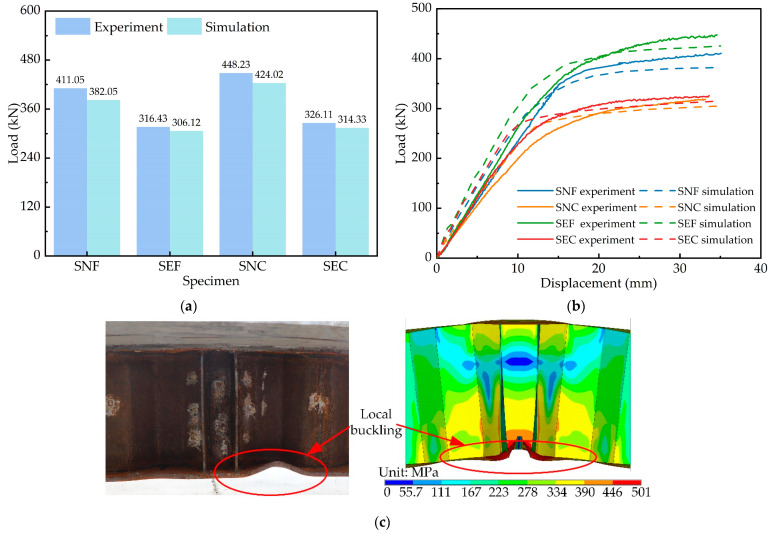
Comparison of specimen and finite element models: (**a**) flexural bearing capacity; (**b**) load–displacement curves; (**c**) failure modes.

The accuracy of the numerical model is analysed by the simulated displacements, ultimate loads, and failure modes. Figure 13a shows the error between the experimental and numerical simulated ultimate loads. The average error is 4.8%, with a standard deviation of 1.52%. Figure 13b plots the load–displacement curves for the experiment and the simulation, with a relatively good overlap between the two curves. In addition, the failure modes of the numerical simulation and the experiment are the same, with local buckling occurring in the bottom flanges as shown in Figure 13c. In general, the results of the numerical simulations are in an acceptable range, which indicates that the numerical model can accurately simulate the failure of the composite girder.

### 4.3. Influence of ECC Slab

As analysed in the previous section, the flexural bearing capacity of the steel-ECC composite girder with corrugated webs is higher than that calculated using the code. From the mechanical analysis, it is known that the tensile stress in the ECC slab is not only related to the ECC strength but also to the cross-sectional dimensions of the ECC slab. Therefore, in this section, to better verify this phenomenon, a parametric analysis of the ECC slab strength, thickness and width is conducted, and the effect of the ECC slab on the mechanical properties of the composite girder is investigated.

#### 4.3.1. ECC Strength

**Figure 14:**
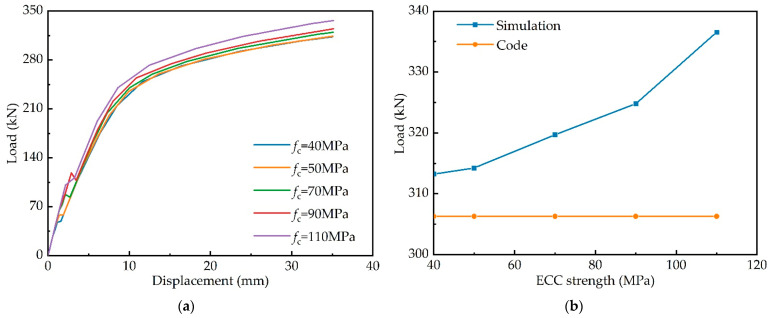
Effects of ECC strength: (**a**) load–deflection curves; (**b**) comparison of flexural bearing capacity.

Six composite girders with different ECC strengths are simulated. Numerical simulations are carried out from low-strength ECC to high-strength ECC and then to ultra-high-strength ECC. In Figure 14a, the three load-displacement curves largely overlap as the ECC strength increases from 40 to 50 MPa. This indicates that low strength ECC has little effect on the flexural capacity. However, the stiffness and flexural capacity increase when the strength increases to 70 MPa. The bearing capacity and stiffness of the structure increase significantly by 8.84% when the strength is up to 110 MPa. Figure 14b compares the flexural capacity obtained by the code and the simulation. It is evident that the flexural capacity calculated with the code is lower than the simulation. When the ECC strength is low, there is little difference between the two. As the strength of the ECC increases, the bearing capacity can no longer be accurately calculated with the code.

#### 4.3.2. Slab Thickness

**Figure 15:**
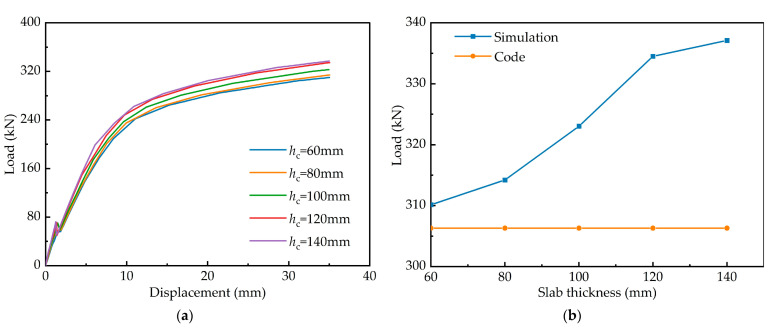
Effects of slab thickness: (**a**) load–deflection curves; (**b**) comparison of flexural bearing capacity.

Finite element models with slab thickness of 60–140 mm and increments of 20 mm are established to analyse the effect of slab thickness on flexural performance. It is clear from Figure 15a that an increase in the thickness of the ECC slab leads to an increase in the bearing capacity and stiffness of composite girders. The difference between the simulated and calculated flexural capacity of each specimen can be seen in Figure 15b. The calculated values of the flexural bearing capacity are on the conservative side and are, on average, 5.71% lower. Compared to the specimen SEC with a slab thickness of 80 mm, the bearing capacity is increased by a maximum of 7.29%.

#### 4.3.3. Slab Width

**Figure 16:**
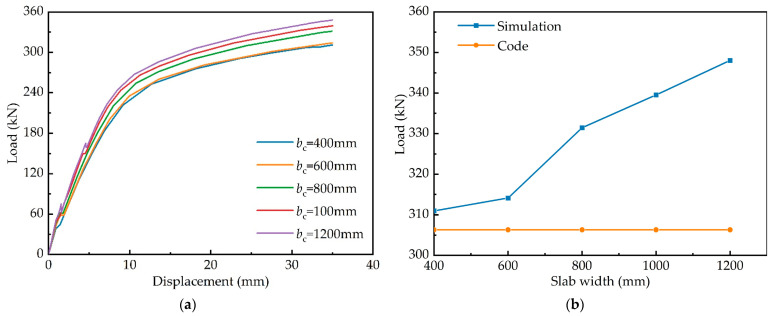
Effects of slab width: (**a**) load–deflection curves; (**b**) comparison of flexural bearing capacity.

The ECC slab width in the finite element model was varied from 400 to 1200 mm to investigate the effect of slab width on structural performance, as illustrated in Figure 16. As the slab width increases, the composite girder’s bearing capacity and stiffness increase. The effect on the structure is slight when the slab width is increased from 400 to 600 mm, but when the slab width is increased to 800 mm, the bearing capacity and stiffness increase rapidly. Compared with the predicted values of the Chinese code, the bearing capacity increases by a maximum of 11.92%.

## 5. Conclusions

In this paper, the mechanical properties of the steel-ECC composite girder with corrugated webs in the negative moment region are investigated through experiments and numerical analysis. The following conclusions are obtained.

(1) The failure modes of all specimens are the typical bending failure. The main failure phenomena are the cracking of the concrete slab and the buckling of the bottom flange of the steel girder. The steel girder and concrete slab work cooperatively during the whole loading process, and no significant slip occurs;

(2) Compared with composite girders with flat webs, the stress of each component of composite girders with corrugated webs is clear. In composite girders with corrugated webs, the web mainly bears the shear force, and the flanges mainly bear the negative moment. Its unique accordion effect makes the strain distribution conform to the “quasi plane assumption” assumption and reduces the structure’s bearing capacity by 25.12%;

(3) The use of ECC instead of NC as the bridge deck slab has, on the one hand, substantially improved the cracking performance of the structure and improved the crack control capability; on the other hand, the application of ECC has increased the flexural bearing capacity and stiffness by 6.04% and 10.54%, respectively. This indicates that the ECC slab affects the flexural bearing capacity of the structure; and

(4) The influence of ECC slab material and dimensional parameters on the bearing capacity is analysed by numerical simulation. The results show that with the increase in ECC material strength and ECC slab section size, the flexural bearing capacity of the composite girders gradually increases. The calculation results with the code tend to be conservative. Therefore, the contribution of the concrete slab to the bending resistance calculation in the negative moment region cannot be ignored.

## Figures and Tables

**Table 1:** Design parameters of the specimens.

Specimens	Concrete Type	Concrete Slab (*b*_c_ × *h*_c_)	Web Type	Steel Girder (*h*_w_ × *ω* × *t*_w_ × *t*_f_)
SNF	NC	600 × 80	Flat web	250 × 150 × 8 × 10
SNC	NC		Corrugated web	
SEF	ECC		Flat web	
SEC	ECC		Corrugated web	

Note: *b*_c_ and *h*_c_ are the concrete slab width and thickness. *h*_w_ and *t*_w_ denote the web height and thickness. *ω* and *t*_w_ represent the flange width and thickness. All dimensions are in mm.

**Table 2:** Mechanical properties of steel plates and steel bars.

Materials	Yield Strength *f*_y_ (MPa)	Ultimate Strength *f*_t_ (MPa)	Elastic Modulus *E*_s_ (GPa)
Steel plates	375.3	501.4	209.8
Steel bars	388.2	493.5	212.3

**Table 3:** Mechanical properties of concrete.

Materials	Nominal Strength *f*_N_ (MPa)	Cube Compressive Strength *f*_cu_ (MPa)	Axial Compressive Strength *f*_ax_ (MPa)	Tensile Strength *f*_tu_ (MPa)	Elastic Modulus *E*_c_ (GPa)
NC	50	61.78	41.4	3.05	37.53
ECC	50	60.99	50.3	4.63	19.60

**Table 4:** Comparison of calculated and experimental bearing capacity.

Specimens	*P*_code_ (kN)	*P*_exp_ (kN)	*P*_code_/*P*_exp_
SNC	306.31	316.43	96.80%
SEC	306.31	326.11	93.93%

Note: *P*_exp_ is the experimental bearing capacity, and *P*_code_ denotes the calculated bearing capacity with the code.

## Data Availability

Data is contained within the article.
